# Characterization of Fasciculation Potentials (FPs) in Amyotrophic Lateral Sclerosis (ALS) and Peripheral Nerve Hyperexcitability Syndromes (PNH)

**DOI:** 10.1155/2021/6631664

**Published:** 2021-05-03

**Authors:** Hua Wang, Bin Liu, Jiyou Tang

**Affiliations:** ^1^Department of Neurology, Shandong Provincial Hospital, Cheeloo College of Medicine, Shandong University, Jinan 250021, China; ^2^Department of Neurosurgery, Shandong Provincial Hospital Affiliated to Shandong First Medical University, Jinan 250021, China; ^3^Department of Neurology, Shandong Qianfoshan Hospital, Cheeloo College of Medicine, Shandong University, Jinan 250014, China

## Abstract

This study is aimed at investigating the features of fasciculation potentials (FPs) in amyotrophic lateral sclerosis (ALS) and peripheral nerve hyperexcitability syndromes (PNH). Needle electrophysiologic examination (EMG) was performed for 5-15 muscles in the ALS and PNH patients. The spontaneous activity of fasciculations and fibrillations/sharp-waves (fibs-sw) was recorded. The distribution, firing frequency, and waveform parameters of FPs in muscles were calculated and compared. In total, 361 muscles in ALS patients and 124 muscles in PNH patients were examined, with the FP detection rates of 45.1% and 53.2%. Moreover, the ALS patients with the upper limb onset had the highest FP detection rate. Fasciculations occurred more frequently in the upper limbs than in the lower limbs in ALS and PNH. The detection rate of fibs-sw in the bulbar muscle was relatively low, which could be elevated when combining fibs-sw and FPs. Benign FPs in PNH were of smaller amplitude, shorter duration, and fewer phases/turns, compared with malignant FPs in ALS. The FP area in PNH was significantly smaller than that in ALS. The incidence of polyphasic FPs in ALS was distinctly greater than that in PNH. The firing frequency of FPs in PNH was higher than that in ALS. There was no significant difference in the amplitude, duration, phases and turns, and area of FPs between groups with and without fibs-sw in the muscles of normal strength in ALS. Conclusively, it is necessary to detect the FPs in the thoracic and bulbar muscles of patients suspected having ALS. FP parameters in ALS are significantly different from PNH.

## 1. Introduction

Fasciculations have long been recognized as a dominant characteristic and early feature of amyotrophic lateral sclerosis (ALS), which can be detected in a wide range of muscles in the body of ALS patients. However, fasciculation potentials (FPs) can indicate ALS only when combined with the progressive limb weakness, atrophy, and/or increased tendon reflexes from the physical examination and neurogenic defects from the electrophysiologic examination (EMG) [1]. Not all affected muscles in ALS patients can display FPs, and not all muscles with FPs are associated with neurogenic damages. Normal muscles in some patients may develop FPs [2], which also could be frequently detected in the peripheral nerve hyperexcitability syndromes (PNH) [3].

PNH includes a series of diseases such as the cramp-fasciculation syndrome, benign fasciculation syndrome (BFS), Isaac's syndrome, and Morvan syndrome. Spontaneous discharges (including fasciculations, doublets, triplets, or multiplets), as well as myokymic discharges and myotonic discharges, which are usually detected by the needle EMG in muscles of PNH patients [4, 5]. Patients with symptoms of muscle twitches and cramps who may suffer from chronic fatigue syndrome (CFS) would struggle to get a diagnosis for years or come to the department of neurology because of the symptom of fasciculations. According to the Awaji criteria proposed in 2008 [6], fasciculations and fibrillations/sharp-waves (fibs-sw) should share similar and equivalent importance in the diagnosis in ALS with EMG. Thus, the detection and observation of FPs should be highlighted during EMG. However, there are few articles describing the prevalence of FPs in needle EMG for ALS and PNH.

In ALS, FPs are expected to have a more complex, instable morphology, which may be involved in normal muscles of a clinically unrelated body region [6]. Nevertheless, it has been reported to be difficult to distinguish the benign and malignant fasciculations just based on the waveform [7]. Concerning the detection rates of malignant and benign FPs, a previous study has revealed that the firing rates in three patients with BFS in the upper limb muscles were higher than those with ALS [8]. However, a recent article has shown an opposite result that the FP firing frequencies in the muscles of ALS patients were significantly higher than those of the PNH patients [9]. In this study, the prevalence, firing pattern, and waveforms of FPs in ALS and PNH were investigated. How the clinicians could benefit of the detection of FPs was studied and analyzed.

## 2. Patients and Methods

### 2.1. Study Subjects

A total of 55 patients, including 36 patients with ALS and 19 patients with PNH, were enrolled in this study. The 36 patients with ALS were admitted to the EMG Laboratories of Shandong Provincial Hospital, from April 2018 to June 2019. In addition, the 19 patients with PNH were admitted from July 2017 to June 2019. All of the 36 patients with ALS had a progressive history of muscle weakness and/or muscle atrophy in one or more body regions, with/without muscle stiffness, for at least 4 months. Complete neurological examination and electromyography were performed. According to the findings from the laboratory and imageological examinations, patients with diabetes, focal radicular neuropathy, multifocal motor neuropathy, cervical spondylosis, and syringomyelia were excluded. There were three patients showing rapid progress in the EMG follow-up within six months, and the data of their last examination were included in the study. Ultimately, according to the revised El Escorial and the Awaji criteria [6, 10], 35 of the recruited patients were classified as probable or definite ALS, while only 1 patient as unclassified. The disease onset occurred in the upper limb in 21 patients, lower limb in 8 patients, bulbar in 5 patients, and respiratory onset (spinal origin) in 2 patients. The syndromes of PNH included the muscle twitches, cramps, stiffness, and myokymia, as well as wide spread of fasciculations. Moreover, the pseudomyotonia and muscle hypertrophy would also be observed. Some of the patients with PNH were accompanied by the hyperhidrosis, insomnia, hallucination, memory loss, epilepsy, and pathological pain, due to the immunological basis associated with antibodies to VGKCs and AChRs. Moreover, in these 19 patients with PNH, three exhibited transient weakness of the limbs and body muscles, and the laboratory tests showed hypokalemia. One of these 3 patients also showed the hypocalcemia and hypermagnesemia due to the electrolyte disorder caused by the Sjogren's syndrome. In one patient complaining of memory loss, seizure, continuous muscle twitching, and coxal muscle spasm, the leucine-rich glioma-inactivated protein1 associated with limbic encephalitis was detected. In addition, two out of the CFS patients were combined with the mild L5-S1 radiculopathy in the lower limbs according to the EMG examination, and however, only the fasciculations in the upper limbs were included herein for the analysis of FP morphology. There was another patient of CFS with a history of mild hoarseness and dysphagia for 2 years, who was enrolled in the Emergency Ward, with hypoxemia due to acute laryngeal paralysis before the EMG examination. Moreover, one patient with CFS for more than 20 years had a mild sensory nerve defect. Another patient with Isaacs' syndrome had a significant reduction in the widespread muscle twitching due to pregnancy. Only one subject of the 19 PNH patients had suffered from kidney cancer for 4 years. Patients in the PNH group had different disease durations, and the patients diagnosed with Isaacs' or Morvan syndrome had relatively shorter symptom histories (with the mean value of 1.8 m) than those diagnosed with CFS/MFS (with the mean value of 34.6 m).

All subjects gave their informed consents, and the protocol was approved by the local research ethic committee.

### 2.2. EMG Detection

All of the 55 patients underwent the accurate neurological examination before the EMG detection, including the LMN related muscle force (MRC scales 0-5), sign of atrophy, and distribution of fasciculations in 4 body regions. Besides, the tendon reflexes and muscle spasticity, plantar response, Hoffman sign, and jaw reflex were also evaluated, which might indicate the UMN functional defect. The disease severities in the patients with ALS were evaluated by the revised ALS Functional Rating Scale.

Needle electromyography and nerve conduction using the electromyographic machine (MEB 9200K, NIHONKOHDEN) were performed, with the filter of 10-5 kHz. The skin temperature was maintained greater than 32°C. At least 6 nerves of the upper and lower limbs were examined, and all the patients were excluded from the significant sensory nerve damages and motor nerve conduction block. Then, 8-15 muscles (a total of 361) in each ALS patient and 5-9 (a total of 124) muscles in the PNH group were subjected to the test for spontaneous activity of fasciculations and fibs-sw. The frequently examined muscles in different body regions in each patient included the tongue, sternocleidomastoid, upper trapezius, deltoid, biceps brachialis, extensor digitorum communis, first dorsal interosseous, abductor pollicis brevis, rectus abdominis, 8^th^ and 10^th^ thoracic paraspinal, iliopsoas, vastus medialis, tibialis anterior, gluteus maximus, and abductor hallucis. The actual number and range of muscles examined were adjusted by the distribution of fasciculations and the weakness complained by the patient. Most patients could cooperate with the needle EMG examination and kept the examined muscles relaxed.

fibs-sw and fasciculations (especially FPs) were explored in at least 6 sites, lasting for at least 120 s for each muscle. The fibs-sw was defined as the regularly firing potentials (duration < 5 ms and amplitude < 1 mV) that lasted for more than 3 s, and the fasciculations were recognized as the motor unit potential-shaped potentials with more than 50 *μ*V, firing in a high irregular pattern. FPs showing not only once were included for the firing frequency calculation (total of 75), and the detection rate of FPs in each muscle was calculated. All the FPs detected (783 in the ALS group and 183 in the PNH group) were saved off line for the further analysis and comparison of amplitude, duration, phases, turns, and area. Complex or malignant FPs were defined as those having either more than 4 phases or increased amplitude and/or duration, compared to normal values of motor unit potentials in the specific muscle. Due to the fact that the data of FPs from different muscles were included herein, it was difficult to analyze and compare the duration and amplitude and area of the FPs, with the normal muscles. Therefore, complex FPs were simply defined as the FPs with more than 4 phases herein. Combined FPs were also noticed, which were however not analyzed separately.

### 2.3. Statistical Analysis

The FPs detected in the ALS patients were divided into 3 groups according to the muscle strength: G1, normal muscle strength (MRC5) group; G2, slightly weak muscle (MRC4) group; and G3, moderately or severely weak muscle (MRC3 and below) group. The FPs detected in the muscles of normal strength in the ALS patients were separately analyzed according to the presence or absence of fibs-sw in the muscle.

Statistical analysis was performed using the SPSS 22.0 software (IBM, USA). Pearson *χ*^2^ test was used to analysis the difference in the gender composition between the PNH and ALS patients. The Mann–Whitney *U* test and Kruskal Wallis test were used to analyze the FP amplitude, duration, area, phases, turns, and firing frequencies between the ALS subgroups and PNH group. The polyphasic FP ratio between groups was compared using the contingency tables. *p* < 0.05 was considered statistically significant.

## 3. Results

### 3.1. Distribution and Detection Rates of FPs

**Figure 1:**
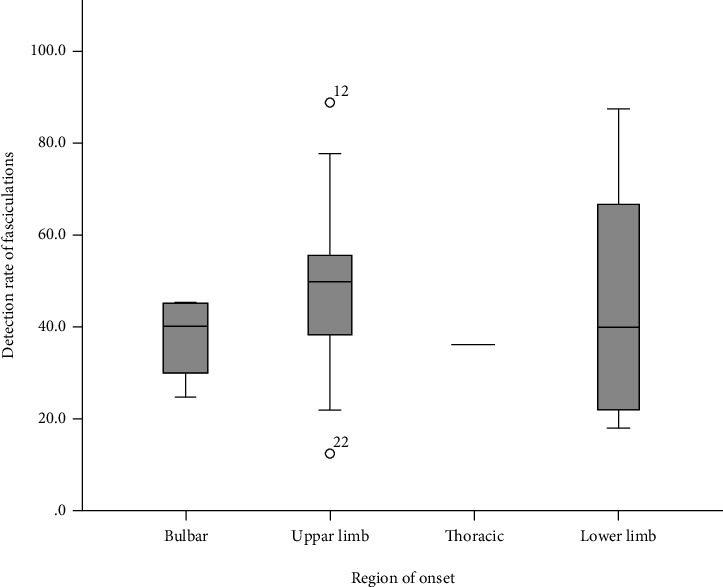
Fasciculation detection rates for ALS classified by an onset site.

In total, 36 patients with ALS and 19 patients with PNH were recruited herein. Spontaneous potentials (FPs and fibs-sw) of 361 muscles in the ALS group and 124 muscles in the PNH group were analyzed (Table 1). The distribution and detection rates of FPs were analyzed and compared. Our results showed that the total detection rates of fasciculations were 45.1% and 53.2% for the ALS and PNH groups, respectively (*p* < 0.001). However, the average number of muscles involved in the detection was less in the PNH group (6.87) compared with the ALS group (10.38) (*p* < 0.001). Classified based on the onset region, the upper limb onset group of ALS had the highest FP detection rate (49.64%), similar to the lower limb onset group (46.50%), both of which were higher than the bulbar and thoracic onset group in the ALS patients (Figure 1).

**Figure 2:**
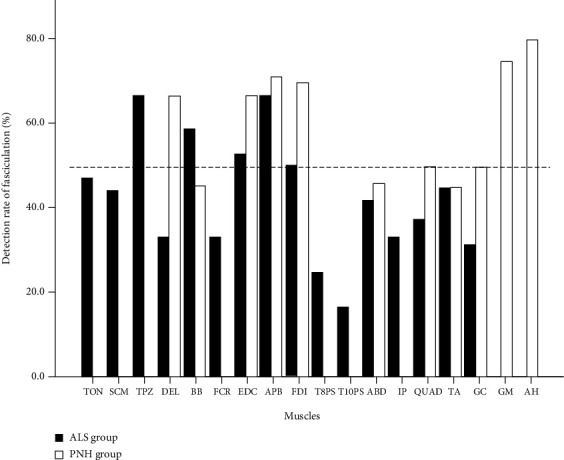
Fasciculation detection rates by needle EMG on each muscle. The fasciculation detection rates by needle EMG on each muscle were analyzed and compared between the ALS and PNH groups. ALS: amyotrophic lateral sclerosis; PNH: peripheral nerve hyperexcitability syndrome; TON: tongue; SCM: sternocleidomastoid; TPZ: upper trapezius; DEL: deltoid; BB: biceps brachialis; FCR: flexor carpi radialis; EDC: extensor digitorum communis; FDI: first dorsal interosseous; APB: abductor pollicis brevis; ABD: rectus abdominis; T8PS and T10PS: 8^th^ and 10^th^ thoracic paraspinal; QUAD: quadriceps femoris; IP: iliopsoas; TA: tibialis anterior; GM: gluteus maximus; AH: abductor hallucis.

**Figure 3:**
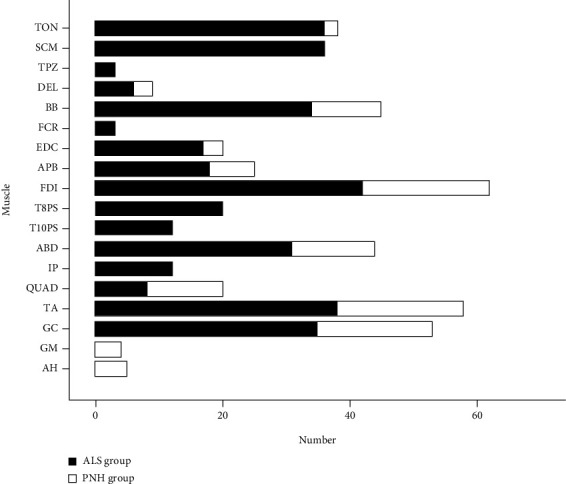
Analysis of examined muscle numbers. The numbers of muscles examined were analyzed and compared between the ALS and PNH groups. ALS: amyotrophic lateral sclerosis; PNH: peripheral nerve hyperexcitability syndrome; TON; tongue; SCM: sternocleidomastoid; TPZ: upper trapezius; DEL: deltoid; BB: biceps brachialis; FCR: flexor carpi radialis; EDC: extensor digitorum communis; FDI: first dorsal interosseous; APB: abductor pollicis brevis; ABD: rectus abdominis; T8PS and T10PS: 8^th^ and 10^th^ thoracic paraspinal; QUAD: quadriceps femoris; IP: iliopsoas; TA: tibialis anterior; GM: gluteus maximus; AH: abductor hallucis.

The detection rates of FPs in each muscle were analyzed and compared between the ALS and PNH groups. As shown in Figure 2, the detection rates of FPs in the lower limb muscles, rectus abdominis, and most of the upper limb muscles in the PNH group were slightly higher than those in the ALS group. The muscles examined with the FP detection rates that were approximately equal to or greater than 50% included the upper trapezius, biceps brachialis, extensor digitorum communis, abductor pollicis brevis, and first dorsal interosseous in the ALS group and the deltoid, extensor digitorum communis, abductor pollicis brevis, first dorsal interosseous, tibialis anterior, gluteus maximus, and abductor hallucis in the PNH group. Fasciculations occurred significantly more frequently in the upper limbs than in the lower limbs in both the ALS and PNH groups. The mean FP detection rates in the upper and lower limbs were 54.7% and 34.6% in the ALS group, which were 63.6% and 52.5% in the PNH group. Fasciculations occurred slightly more often in the proximal extremity (median, 55%) than the distal muscles of upper limb (median, 51.7%). Meanwhile, they were more often detected in the distal extremities (median, 70%) than in the proximal upper limb muscles (median, 50%). Although the detection rates of FPs in some muscles (e.g., in the upper trapezius, deltoid, gluteus maximus, and abductor hallucis) were higher than 60%, the number of these muscles examined in this study was actually not high (Figure 3).

**Figure 4:**
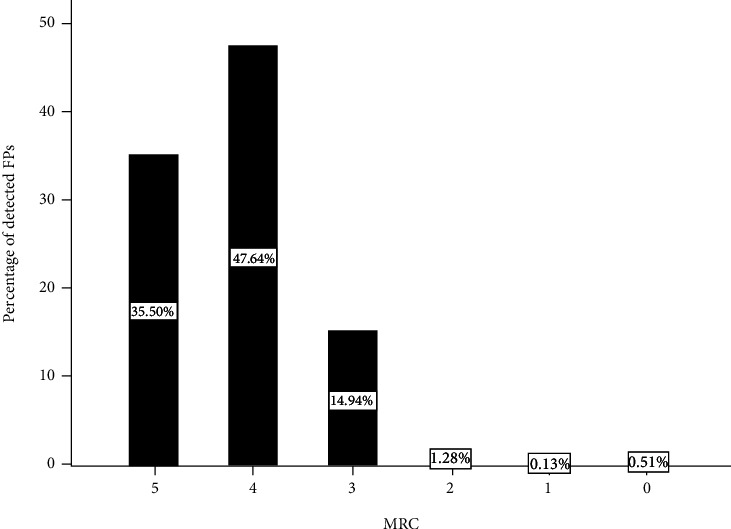
Percentage of FPs detected in different muscle strength groups. The number and percentage of FPs detected in the different muscle strength groups in all FPs in ALS were analyzed. MRC: medical research council.

Fasciculations were more easily detected in the slightly weak muscles (MRC4, 47.6%) in the ALS patients than in the normal strength muscle (MRC5, 35.5%). The incidence of FPs in the moderately or severely weak muscles (MRC3 and below, 16.9%) was significantly lower than that in the normal strength or slightly weak muscles (Figure 4).

**Figure 5:**
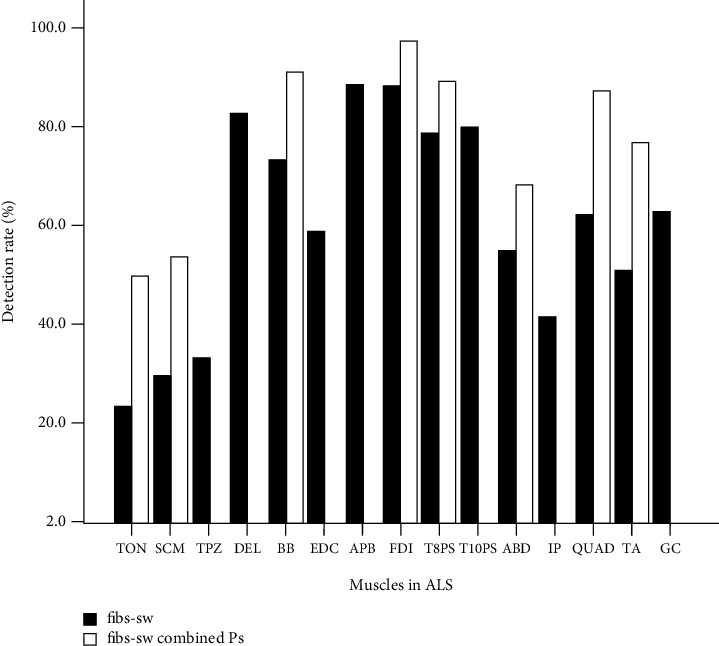
Detection rate of spontaneous activity combined with fibs-sw and FPs in each muscle examined in ALS. fibs-sw: fibrillations/sharp-waves; FPs: fasciculation potentials; ALS: amyotrophic lateral sclerosis; PNH: peripheral nerve hyperexcitability syndrome; TON: tongue; SCM: sternocleidomastoid; TPZ: upper trapezius; DEL: deltoid; BB: biceps brachialis; EDC: extensor digitorum communis; FDI: first dorsal interosseous; APB: abductor pollicis brevis; ABD: rectus abdominis; T8PS and T10PS: 8^th^ and 10^th^ thoracic paraspinal; QUAD: quadriceps femoris; IP: iliopsoas; TA: tibialis anterior.

The fibs-sw at more than two sites examined in the muscle by the needle EMG was considered abnormal. The detection rate of fibs-sw in the bulbar muscle was relatively low (tongue 23.7% and sternocleidomastoid 29.7%). Given the difficulty of patient coordination in the muscle examination of bulbar muscles, it was impossible to distinguish the fibs-sw due to the incomplete relaxation, in about 10% (or less) of the examined lingual muscles. On the other hand, the FPs of the bulbar muscle were more easily to detected, with the detection rates of 42.1% and 44.4%, respectively, for the tongue and sternocleidomastoid. There were 8 and 9 more patients showing the FPs in the bulbar muscles without denervation activity. Therefore, combined with the spontaneous activity of FPs and fibs-sw, the whole neurogenic detection rate of the bulbar muscles could be significantly improved, approximately 50.0% and 54.1% for the tongue and sternocleidomastoid, respectively. Although the biceps had a positive rate of 73.5% of fibs-sw, there were another 6 muscles showing FPs, and a combined 91.2 neurogenic activity was accomplished. The same phenomenon occurred in the anterior tibialis, covering from 51.3% to 71.9% (Figure 5).

**Figure 6:**
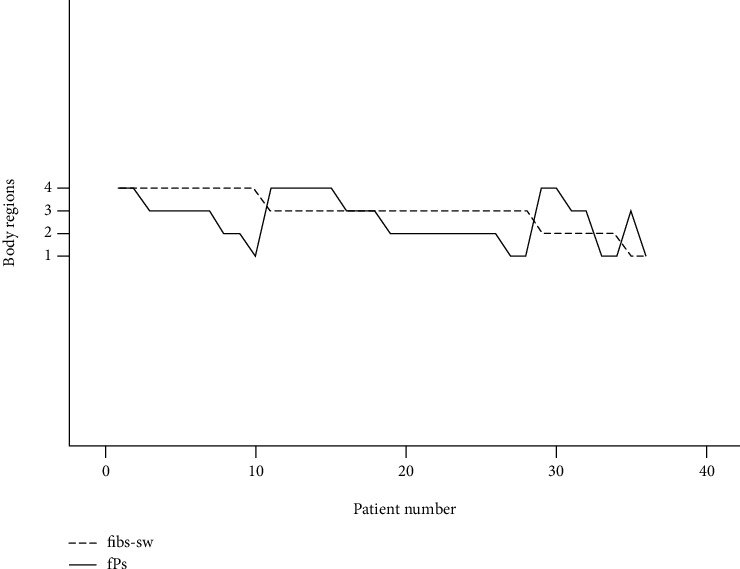
Comparison of number of involved body regions of fasciculation potentials (FPs) and fibrillations/sharp-waves (fibs-sw) in ALS.

In general, the distribution of FPs in each body region was not consistent with the fibs-sw. When the denervation activity occurred in no more than 3 individual body regions, the distribution of FPs was more extensive than fibs-sw, with an incidence of 47.8% (11/23). However, when the fibs-sw was widely distributed (3 or 4 body regions), the distribution range of FPs was decreased correspondingly. In the body regions not clinically affected, the FPs and denervation activities could be examined. Our results showed that 63.9% (23/36) of the patients showed fibs-sw or FPs in the body regions not clinically involved (Figure 6).

### 3.2. Characterization of FPs

**Figure 7:**
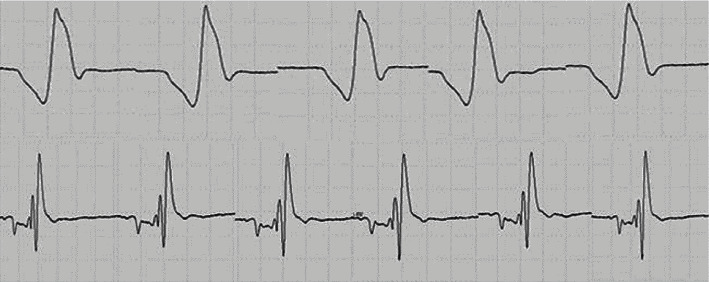
Fasciculations recorded in tibialis anterior muscle in PNH.

**Figure 8:**
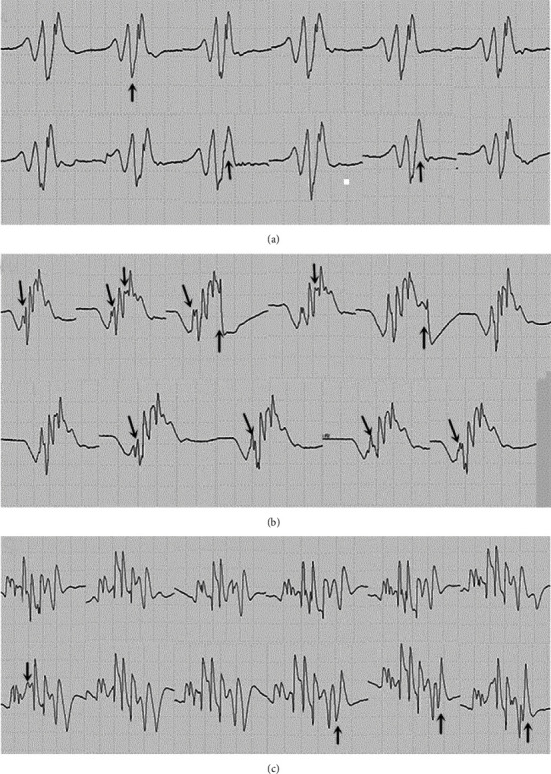
Fasciculations recorded in ALS. (a) Fasciculations recorded in the biceps (MRC3, fibs-sw 2+) of an ALS patient. (b, c) Fasciculations recorded in the rectus abdominis (MRC5, fibs-sw 2+) of another ALS patient. The components showing intermittent conduction block and multifocal triggering were marked by arrows.

The shape and frequency of FPs discharging more than once were observed. Repeated released FPs in the PNH group were generally simple and stable (Figure 7). However, most the FPs repeatedly recorded in the ALS group showed morphological diversity, complexity, and instability (Figure 8). Besides, the rate of stable FPs in the ALS group was 34.2%, compared with the rate of almost 100% in the PNH group, which was higher in the muscles without fibs-sw (43.75%) of the ALS patients.

### 3.3. Comparison of Fasciculations in ALS and PNH

Benign FPs in the PNH group were with smaller amplitude (*p* < 0.001), shorter duration (*p* < 0.001), and fewer phases and turns (*p* < 0.001), when compared with the malignant FPs in ALS. The area of FPs in the PNH group was significantly smaller than that in the ALS group (*p* < 0.001). The incidence of polyphasic FPs (phases > 4) in ALS was distinctly greater than PNH (*p* < 0.01). The firing frequency of FPs repeatedly recorded in the PNH group was higher than that in the ALS group (Table 2).

A total of 278 FPs were recorded in the normal strength muscles of ALS, which were divided into two groups (i.e., the G1 and G2 groups), according to whether they were accompanied by the onset of denervation activities. There was no significant difference in the amplitude, duration, phases and turns, and area of FPs between the G1 and G2 groups in ALS. However, the proportion of the polyphasic FPs in the G2 group was significantly higher than that in the G1 group (*p* < 0.001). However, the amplitude, duration, phases and turns, area, and polyphasic ratios of FPs in the normal strength muscles without denervation in the ALS group (G1) showed the same diversity as those between the ALS and PNH groups (Table 3).

The number of fasciculations detected in the muscles of different muscle strength varied greatly. The G2 group with mild muscle weakness of ALS had the highest number of FPs, followed by the G1 normal muscle group, and the lowest number of FPs was observed for the G3 severe muscle weakness group. The mean number of fasciculations (of different shapes) of each muscle showed the same trend, with the mean shapes of 7.72, 8.67, and 7.03, respectively, for the G1, G2, and G3 groups, with however no statistically significant difference (based on Fisher's exact test). The amplitude of FPs in the ALS G2 group (MRC4) was higher than those in the normal and severe weak muscles (for both the G1 and G3 groups, *p* < 0.05), and the area of FPs in the G2 group was larger than that in the G1 group (*p* < 0.05). The duration of FPs in various ALS groups did not differ significantly from each other nor did the phases and turns of FPs. The percentage of polyphasic FPs was higher in the groups of muscles with normal strength and mild weakness (*p* < 0.001). Accompanied with the greater FP number in the mild weak muscle group (G2), the FP firing frequency of the G2 group was higher than that in the G1 group, which was the lowest in the G3 group, with however no statistically significant difference (Table 4).

## 4. Discussion

In this study, the whole detection rate of FPs in the ALS patients with the needle EMG was 45.1%, which was significantly lower than the detection rate with ultrasound (74%) [9]. Indeed, the application of the needle EMG examination has been limited by the detection range of the concentric needle electrodes. Regensburger et al. [11] have reported that the mean detection radius of EMG for the fasciculations was 7.75 mm, comparable to a motor unit size. However, considering the irreplaceable importance of needle EMG in the detection of neurogenic defects, combined with the significance of FPs as the denervated potential in the electrophysiological diagnosis of ALS, it is necessary to detect and identify the FPs in the thoracic and bulbar regions of patients, where the denervated potentials are difficult to detect accurately. In this study, we routinely examined the lingual and sternocleidomastoid muscles of the medulla oblongata segment, paravertebral muscles, and rectus abdominis of the thoracic segment of ALS patient. The sensitivities of the detection for neurogenic deficiency associated with combined FPs and fibs-sw were increased to 67.8% and 96.6% in the bulbar and thoracic regions, higher than those of the ultrasonic detection of a single muscle in these regions [9, 12].

It is difficult to distinguish ALS from PNH by examining the FP detection rate in the muscles of a particular body region of the patient. Our results showed that the FP detection rate in the PNH group (53.2%) was higher than that in the ALS group. The patients in the PNH group could be divided into two groups (i.e., the groups with disease durations less or more than 6 months), and the mean detection rates of FPs in these two groups were 70.47% and 47.5%, respectively, with no statistical differences in PNH between these two groups. The detection rates were both higher than those in the ALS patients. However, patients with PNH tended to be associated with a tremor in the extremities and gluteal muscle symptoms (especially the patients with subacute onset), where the FPs companied with neuromyotonic and myokymic discharges would be easily detected and the fasciculations in two sides of the tongue were rarely observed.

The FPs in ALS were generally more complex than those in PNH. In this study, our results showed that there were significant differences in the amplitude, duration, phases, and turns and area of FPs between these two groups. Although the polyphasic FP proportion of the PNH group was significantly lower than that of the ALS group, it was still higher when compared with the previous findings [13]. Several influencing factors should be also considered. Firstly, two patients with hypokalemia in the PNH group had proportions of polyphasic FPs up to 45%-50% according to the retrospective study. Considering the acute onset of muscle twitches, spasm and normal serum creatine enzyme levels, the FPs detected in those two patients were not excluded. In one of these two patients, the typical after-discharge potentials were observed during the motor nerve conduction test, and neither neurogenic nor myogenic MUPs were observed during the weak effort analysis, for both of these two patients. Kuwabara and Misawa [14] have found that the axons of motor nerve became substantially hyperpolarized in the patient with hypokalemia. Therefore, we suggest that the hypokalemia and transient muscle weakness are merely companying symptoms, which might be due to the expression of related antibodies in the kidney [15]. However, the minor muscle damages that could not be detected by needle EMG could not be excluded, which may be one of the reasons for the increased polyphasic FP ratio.

de Carvalho and Swash [13] have revealed that the FPs in the ALS patient muscles show no reinnervation (the earliest stage of that muscle), which are similar with FPs in the BFS in size, duration, and morphology. In this study, our results showed the FPs recorded in the muscles with normal strength, and no fibs-sw were of significantly higher amplitude, longer duration, and increased number of phases, compared with the FPs in the PNH group. However, the MUP morphology of different muscles was not quantitatively analyzed, and it was possible that the FPs with more complex morphology and longer duration were accompanied by the neurogenic changes of MUPs in the muscles with normal strength and no fibs-sw [16]. In addition, these results supported the findings from Krarup [16], showing that, in 44% of muscles in the ALS patients, the neurogenic MUPs occurred in the absence of the denervation activity. Our results showed that, in patients with ALS, the FPs with the normal morphology appear first, followed by the FPs with more complex patterns and the subsequent occurrence of denervation activity (fibs-sw), which were in line with the findings from de Carvalho and Swash [13]. If combined with the pathological evolution of patients with ALS, the fibs-sw would not disappear, once it appeared during the short-medium electrophysiological follow-up. In general, the earlier the course of ALS, the more difficult it is to detect the denervation potentials, while the more progressed the disease course, the more difficult it is to detect the fasciculation potentials.

## 5. Conclusion

In summary, our results showed that the firing frequencies of FPs with the same morphology were higher in the PNH group than in the ALS group, which was contrary to some previous findings [9]. However, further studies are still needed to verify whether the FPs detected at the same site had the same shape on the needle EMG. In addition, the number of included patients was limited, especially for the ALS group with lower limb and bulbar-onset. Only 2 patients had thoracic-onset in our ALS patient group. This had impact on results, as statistical comparisons of FP detection rates between groups defined by region-of-onset lacks the reliability. Thus, further in-depth studies are still needed, with enlarged sample sizes and exclusion screening for the PNH patients.

## Figures and Tables

**Table 1:** Demographic characteristics and fasciculation detection rate in muscles of ALS and PNH.

	ALS (*n* = 36)	PNH (*n* = 19)	*p*
Gender (M : F)	27 : 9	10 : 9	0.388
Age (years)	59.6 (8.2)	54.0 (14.8)	<0.001
Disease duration (month)	15.6 (10.6)	20.0 (31.3)	<0.001
ALSFRS-R	39.8 (5.5)	N/A	N/A
Overall fasciculation detection rate (%)	45.1 (163/361)	53.2 (66/124)	<0.001

The data of age, disease duration, and ALSFRS-R were shown as the mean (SD). ALS: amyotrophic lateral sclerosis; PNH: peripheral nerve hyperexcitability syndrome; and ALSFRS-R: revised ALS Functional Rating Scale.

**Table 2:** Parameters of fasciculation potentials (FPs) in ALS and PNH.

Number of FPs	ALS FPs	PNH FPs	*p*
	783	183	
Amplitude (*μ*V)	508.00 (85-6080)	333.00 (90-2300)	*p* ≤ 0.001
Duration (ms)	15.73 (0.9-30)	13.56 (4.7-27.0)	*p* ≤ 0.001
Number of phases	4.32 (1-16)	3.39 (1-9)	*p* ≤ 0.001
Number of turns	4.25 (0-22)	2.73 (0-10)	*p* ≤ 0.001
Area (mVms)	0.994 (0.1-25.6)	0.49 (0.1-5.2)	*p* ≤ 0.001
Polyphasic (%)	48.15	26.23	*p* ≤ 0.001
FP frequency (Hz)	3.02 (0.3-20.0)	7.15 (5.5-40.0)	0.002

The data were shown as the median (range). ALS: amyotrophic lateral sclerosis; PNH: peripheral nerve hyperexcitability syndrome; MRC: medical research council.

**Table 3:** Parameters of fasciculation potentials (FPs) in ALS with MRC5 and PNH.

Number of FPs	G1 ALS-MRC 5 without partial denervation	G2 ALS-MRC 5 with partial denervation	G3 PNH FPs	*p*
	149	129	183	
Amplitude (*μ*V)	477.50 (95-4681)	423.00 (85-4998)	333.00 (90-2300)	G1 vs. G3, *p* ≤ 0.001 G2 vs. G3, *p* ≤ 0.001 G1 vs. G2, 0.549
Duration (ms)	16.4 (6.3-27.7)	15.48 (5.6-25.6)	13.56 (4.7-27.0)	G1 vs. G3, 0.002 G2 vs. G3, 0.004 G1 vs. G2, 0.740
Number of phases	4.3 (1-10)	4.37 (2-16)	3.39 (1-9)	G1 vs. G3, *p* ≤ 0.001 G2 vs. G3, *p* ≤ 0.001 G1 vs. G2, 0.874
Number of turns	3.91 (0-15)	4.41 (0-16)	2.73 (0-10)	G1 vs. G3, *p* ≤ 0.001 G2 vs. G3, *p* ≤ 0.001 G1 vs. G2, 0.424
Area (mVms)	0.96 (0.1-13.5)	0.95 (0.1-11.0)	0.49 (0.1-5.2)	G1 vs. G3, *p* ≤ 0.001 G2 vs. G3, *p* ≤ 0.001 G1 vs. G2, 0.741
Polyphasic (%)	46.98	51.94	26.23	G1 vs. G3, *p* ≤ 0.001 G2 vs. G3, *p* ≤ 0.001 G1 vs. G2, *p* ≤ 0.001
FP frequency (Hz)	2.50 (0.5-7.8)	2.88 (1.0-8.3)	7.15 (5.5-40.0)	G1 vs. G3, 0.008 G2 vs. G3, 0.002 G1 vs. G2, 0.681

The data were shown as the median (range). ALS: amyotrophic lateral sclerosis; PNH: peripheral nerve hyperexcitability syndrome; MRC: medical research council.

**Table 4:** Parameters of fasciculation potentials (FPs) in ALS with different muscle strength and PNH.

Number of FPs	G1 ALS-MRC 5	G2 ALS-MRC 4	G3 ALS-MRC 3	G4 PNH FPs	*p*
	278	373	132	183	
Amplitude (*μ*V)	445.00 (85-4998)	572.00 (89-4681)	445.50 (40-6080)	333.00 (90-2300)	G1 vs. G4, *p* ≤ 0.001 G2 vs. G4, *p* ≤ 0.001 G3 vs. G4, *p* ≤ 0.001 G1 vs. G2, 0.001 G1 vs. G3, 0.912 G2 vs. G3, 0.006
Duration (ms)	15.73 (5.6-27.7)	15.63 (9-15.0)	16.35 (7.8-27.0)	13.56 (4.7-27.0)	G1 vs. G4, *p* ≤ 0.001 G2 vs. G4, *p* ≤ 0.001 G3 vs. G4, *p* ≤ 0.001 G1 vs. G2, 0.639 G1 vs. G3, 0.243 G2 vs. G3, 0.277
Number of phases	4.34 (1-16)	4.35 (1-16)	4.18 (2-14)	3.39 (1-9)	G1 vs. G4, *p* ≤ 0.001 G2 vs. G4, *p* ≤ 0.001 G3 vs. G4, *p* ≤ 0.001 G1 vs. G2, 0.827 G1 vs. G3, 0.675 G2 vs. G3, 0.529
Number of turns	4.15 (0-16)	4.47 (0-22)	3.90 (1-13)	2.73 (0-10)	G1 vs. G4, *p* ≤ 0.001 G2 vs. G4, *p* ≤ 0.001 G3 vs. G4, *p* ≤ 0.001 G1 vs. G2, 0.128 G1 vs. G3, 0.610 G2 vs. G3, 0.066
Area (mVms)	0.96 (0.1-13.5)	1.04 (0.1-13.3)	0.96 (0.1-25.6)	0.49 (0.1-5.2)	G1 vs. G4, *p* ≤ 0.001 G2 vs. G4, *p* ≤ 0.001 G3 vs. G4, *p* ≤ 0.001 G1 vs. G2, 0.034 G1 vs. G3, 0.333 G2 vs. G3, 0.478
Polyphasic (%)	49.28	48.79	43.94	26.23	G1 vs. G4, *p* ≤ 0.001 G2 vs. G4, *p* ≤ 0.001 G3 vs. G4, *p* ≤ 0.001 G1 vs. G2, *p* ≤ 0.001 G1 vs. G3, *p* ≤ 0.001 G2 vs. G3, *p* ≤ 0.001
FP frequency (Hz)	2.88 (0.5-8.3)	3.60 (0.5-12.5)	1.20 (0.3-20.0)	7.15 (5.5-40.0)	G1 vs. G4, 0.001 G2 vs. G4, 0.004 G3 vs. G4, 0.018 G1 vs. G2, 0.356 G1 vs. G3, 0.542 G2 vs. G3, 0.452

The data were shown as median (range). ALS: amyotrophic lateral sclerosis; PNH: peripheral nerve hyperexcitability syndrome; MRC: medical research council.

## Data Availability

The data that support the findings of this study are available on request from the corresponding author.
